# Comparison of Improvement in Patient-Reported Knee Function After Revision and Multiple-Revision ACL Reconstruction Compared With Primary ACL Reconstruction

**DOI:** 10.1177/23259671231217725

**Published:** 2023-12-22

**Authors:** Janina Kaarre, Zachary J. Herman, Alberto Grassi, Eric Hamrin Senorski, Volker Musahl, Kristian Samuelsson

**Affiliations:** † Department of Orthopaedics, Institute of Clinical Sciences, Sahlgrenska Academy, University of Gothenburg, Gothenburg, Sweden; ‡ Sahlgrenska Sports Medicine Center, Gothenburg, Sweden; § Department of Orthopaedic Surgery, UPMC Freddie Fu Sports Medicine Center, University of Pittsburgh Medical Center, Pittsburgh, Pennsylvania, USA; ‖ IIa Clinica Ortopedica e Traumatologica, IRCCS Istituto Ortopedico Rizzoli, Bologna, Italy; ¶ Unit of Physiotherapy, Department of Health and Rehabilitation, Institute of Neuroscience and Physiology, Sahlgrenska Academy, University of Gothenburg, Gothenburg, Sweden; # Department of Orthopaedics, Sahlgrenska University Hospital, Mölndal, Sweden; Investigation performed at the Department of Orthopaedics, Institute of Clinical Sciences, Sahlgrenska Academy, University of Gothenburg, Gothenburg, Sweden

**Keywords:** anterior cruciate ligament, KOOS, Knee injury and Osteoarthritis Outcome Score, multiple revision, reconstruction, revision

## Abstract

**Background:**

Graft failure after anterior cruciate ligament reconstruction (ACLR) is a debilitating complication often requiring revision surgery. It is widely agreed upon that functional knee outcomes after revision ACLR (r-ACLR) are inferior compared with those after primary reconstruction. However, data are scarce on outcomes after multiple-revision ACLR (mr-ACLR).

**Purpose:**

To compare patient-reported knee function in terms of Knee injury and Osteoarthritis Outcome Score (KOOS) preoperatively and 1-year postoperatively after primary ACLR, r-ACLR, and mr-ACLR and evaluate the pre- to postoperative improvement in KOOS scores for each procedure.

**Study Design:**

Cohort study; Level of evidence, 3.

**Methods:**

Patients from the Swedish National Knee Ligament Registry who underwent their index ACLR between 2005 and 2020 with a minimum age of 15 years at the time of surgery were included in this study. All patients had pre- and postoperative KOOS data. The 1-year postoperative KOOS and the pre- to postoperative changes in KOOS were assessed between patients who underwent primary ACLR and those who underwent subsequent r-ACLR and mr-ACLR.

**Results:**

Of 20,542 included patients, 19,769 (96.2%) underwent primary ACLR, 760 (3.7%) underwent r-ACLR, and 13 (0.06%) underwent mr-ACLR. Patients who underwent r-ACLR had significantly smaller pre- to postoperative changes on all KOOS subscales compared with patients undergoing primary ACLR (*P* < .0001 for all). Furthermore, patients in the mr-ACLR group had significantly smaller changes in the KOOS-Pain subscale compared with patients in the r-ACLR group (–9 ± 23.3 vs 2.5 ± 18; *P* = .024).

**Conclusion:**

The study results indicated that while improvement is seen after primary ACLR, r-ACLR, and mr-ACLR, the greatest improvement in functional outcomes is observed after primary ACLR. Patients who underwent at least 1 r-ACLR, specifically mr-ACLR, had lower postoperative outcome scores, indicating that primary ACLR may provide the best chance for recovery after ACL injury.

Graft failure after anterior cruciate ligament reconstruction (ACLR) is a debilitating complication often requiring revision surgery. It is widely agreed upon that functional knee outcomes after revision ACLR (r-ACLR) are inferior to those after primary reconstruction.^5,39^ However, data are scarce on the outcomes after multiple-revision ACLR (mr-ACLR) as they compare to those after r-ACLR. Research has shown that allograft use, concomitant injury to the medial collateral ligament and meniscus, and increased posterior tibial slope during r-ACLR are risk factors for subsequent failure. At the same time, inferior patient-reported outcomes (eg, Tegner activity level and Lysholm score) after mr-ACLR have also been reported.^1,2,12,37,38^

The few case series and comparative studies presented in the literature report a reasonable rate of return to sports and knee function after mr-ACLR, yet only small percentages of patients return to pre-mr-ACLR activity levels.^11,15,17,19,23^ Furthermore, limited data exist for comparing the improvement of patient-reported knee function pre- to postoperatively between primary ACLR, r-ACLR, and mr-ACLR. Increased knowledge of the possible differences in outcome improvements after primary ACLR, r-ACLR, and mr-ACLR would be important to guide patients through the treatment process, allowing them to access up-to-date information on the expected outcomes.

This study aimed to compare (1) patient-reported knee function in terms of the Knee injury and Osteoarthritis Outcome Score (KOOS) preoperatively and 1 year postoperatively after primary ACLR, r-ACLR, and mr-ACLR as well as (2) the pre- to postoperative change in KOOS scores for each procedure. It was hypothesized that patients with r-ACLR and mr-ACLR would experience less pre- to postoperative improvement than patients with primary ACLR and r-ACLR, respectively.

## Methods

This cohort study was conducted according to the Guidelines for Strengthening the Reporting of Observational Studies in Epidemiology,^14^ and the study protocol received institutional review board approval. The data on the included patients were collected from the Swedish National Knee Ligament Registry (SNKLR). The SNKLR was developed in January 2005 and contains both surgeon and patient-reported data, including, but not limited to, demographic, injury, and surgical characteristics, as well as patient-reported outcome measures. While all data related to descriptive characteristics—including sex, age, body mass index, injury, and surgery (eg, activity at the time of injury, laterality, concomitant injuries, and graft type)—are reported by the surgeon, the patient is asked to complete questionnaires regarding their current knee function, such as the KOOS.^40^ Information on ACL revision surgery is registered separately and merged with data on primary ACLR by using patients’ social security numbers. The registry has previously been described in detail.^24,30^

### Data Collection and Study Sample

We included data on patients at least 15 years old who underwent their index ACLR between 2005 and 2020 and had preoperative and 1-year follow-up outcome data. Patients with previous knee surgery, concomitant fracture, concomitant posterior cruciate ligament, or neurovascular injury were excluded. The study population was divided into 3 different groups: (1) primary ACLR—patients who underwent only 1 ipsilateral ACLR procedure; (2) r-ACLR—patients who underwent a subsequent ipsilateral r-ACLR procedure; and (3) mr-ACLR—patients who underwent at least two ipsilateral r-ACLR procedures.

### Outcome Measures

The primary outcome of interest was the pre- to postoperative change in the KOOS, while the secondary outcome of interest was the 1-year postoperative KOOS. The 1-year postoperative KOOS has previously been reported to be equivalent to the 2-year postoperative KOOS.^28^

The KOOS comprises 42 items grouped within 5 subscales—including Pain, Symptoms, Function in Sport and Recreation (Sport/Rec), Quality of Life (QoL), and Activities of Daily Living (ADL). The patient responses are further scored on a scale from 0 to 4, and each subscale is scored from 0 to 100, where a higher score indicates better outcomes.^27^ Although initially designed for knee osteoarthritis, the KOOS has also been used in other orthopaedic knee conditions, including ACLR, to evaluate outcomes after a specific treatment approach.^27,32^ In addition to the 5 subscales, we included the KOOS-4 score, which is the mean of the KOOS Pain, Symptoms, Sport/Rec, and QoL subscales.

### Statistical Analyses

All statistical analyses were performed using the SAS System for Windows software Version 9.4 (SAS Institute). Counts with proportions were used to present categorical variables, while means with standard deviations and medians with minimum and maximum were used to present continuous and ordinal data, respectively. For comparisons between groups, the Fisher exact test was used for dichotomous variables, while the chi-square test and the Fisher nonparametric permutation test were utilized for nonordered categorical variables and continuous variables, respectively. The confidence interval for the mean difference between groups was based on the Fisher nonparametric permutation test. Effect sizes were calculated using the Cohen *d* when n >50 and the Hedges *g* when n <50, respectively. For comparisons between groups, a *t* test was used for continuous variables, adjusting for preoperative KOOS scores and baseline characteristics. Dropout analyses were performed on the included patients and patients who were excluded because of missing pre- and/or postoperative KOOS data (Supplemental Table S1, available separately). All significance tests were conducted at the 5% significance level (threshold for significance, *P* < .05).

## Results

### Baseline Characteristics

**Figure 1.**
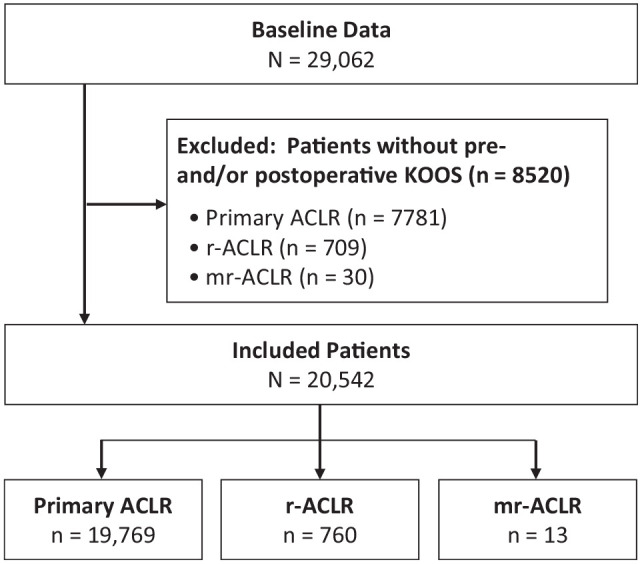
Flowchart of patient enrollment. ACLR, anterior cruciate ligament reconstruction; KOOS, Knee injury and Osteoarthritis Outcome Score; mr-ACLR, multiple-revision ACLR; r-ACLR, revision ACLR.

Out of the 20,542 patients who were included in the study, 19,769 (96.2%) underwent primary ACLR, 760 (3.7%) underwent r-ACLR, and 13 (0.06%) underwent mr-ACLR (Figure 1). Baseline characteristics of the study patients before the most recent ACLR are shown in Table 1. Baseline characteristics before the index ACLR are available separately (Supplemental Table S2).

**Table 1:** Baseline Characteristics of Patients Before Latest ACLR^*a*^

Variable	ACLR (n = 19,769)	r-ACLR (n = 760)	mr-ACLR (n = 13)	Comparison Between Groups: MD (95% CI)	
				ACLR vs r-ACLR	r-ACLR vs mr-ACLR
Age at surgery, y	29.1 ± 10.6 26 (16-74)	25.8 ± 8.2 23 (16-58)	27.8 ± 7.2 28 (20-42)	3.3 (2.5 to 4.0) ***P* = .0020;** ES = 0.312	−2.1 (–6.2 to 3.0) *P* = .36; ES = 0.253
Sex (male)	10,436 (52.8)	395 (52.0)	7 (53.8)	3.3 (2.5 to 4.0) ***P* = .0020**; ES = 0.312	1.9 (–28.1 to 29.3) *P* > .99; ES = 0.04
BMI, kg/m^2^	24.7 ± 3.3 24.3 (15.1-47.1)	24.5 ± 3.0 24.2 (17.9-37.6)	24.5 ± 2.6 23.6 (21.2-29.4)	*P* = .21	−0.0 (–1.5 to 17.8) *P* = .97; ES = 0.008
Activity during injury^*b*^				***P* < .0001**	
Alpine/skiing	3,464 (17.6)	46 (6.1)	0 (0.0)	−11.5 (–13.3 to −9.6) ***P* < .0001**; ES = 0.36	−6.1 (–11.7 to −0.5) *P* = .89; ES = 0.36
Pivoting sport	12,492 (63.3)	468 (61.9)	9 (69.2)	−1.4 (–5 to 2.2) *P* = .45; ES = 0.03	7.3 (–28.2 to 28.6) *P* = .82; ES = 0.15
Nonpivoting sport	861 (4.4)	18 (2.4)	0 (0.0)	−2 (–3.2 to −0.8) ***P* = .0063**; ES = 0.11	−2.4 (–4.5 to 29.4) *P* > .99; ES = 0.22
Other physical activity	795 (4.0)	39 (5.2)	1 (7.7)	1.1 (–0.5 to 0) *P* = .16; ES = 0.05	2.5 (–5.1 to 36.2) *P* > .99; ES = 0.10
Traffic-related	326 (1.7)	7 (0.9)	1 (7.7)	−0.7 (–1.5 to 0) *P* = .14; ES = 0.06	6.8 (–0.6 to 39.9) *P* = .26; ES = 0.34
Other	1,787 (9.1)	178 (23.5)	2 (15.2)	14.5 (11.4 to 17.6) *P* **< .0001**; ES = 0.40	−8.2 (–21.3 to 26.3) *P* = .76; ES = 0.21
Cartilage injury					
Lateral femoral condyle	1,122 (5.7)	72 (9.5)	3 (23.1)	3.8 (1.6 to 6) ***P* < .0001**; ES = 0.14	13.6 (–4.5 to 47.2) *P* = .25; ES = 0.37
Medial femoral condyle	3,677 (18.6)	196 (25.8)	5 (38.5)	7.2 (4 to 10.4) ***P* < .0001**; ES = 0.17	12.7 (–12.6 to 44.1) *P* = .46; ES = 0.27
Lateral patella	661 (3.3)	35 (4.6)	1 (7.7)	1.3 (–0.3 to 2.8) *P* = .084; ES = 0.06	3.1 (–4.5 to 36.4) *P* = .93; ES = 0.11
Medial patella	1,047 (5.3)	43 (5.7)	2 (15.4)	0.4 (–1.4 to 2.1) *P* = 0.71; ES = 0.02	9.7 (–3.4 to 43.5) *P* = .34; ES = 0.32
Lateral tibial plateau	1,360 (6.9)	70 (9.2)	1 (7.7)	2.3 (0.2 to 4.5) ***P* = .020**; ES = 0.09	−1.5 (–9.4 to 32.3) *P* > .99; ES = 0.05
Medial tibial plateau	1,059 (5.4)	56 (7.4)	2 (15.4)	2.0 (0.1 to 4) ***P* = .025**; ES = 0.08	8 (–5.1 to 42) *P* = .51; ES = 0.25
Trochlea	663 (3.4)	45 (5.9)	1 (7.7)	2.6 (0.8 to 4.3) ***P* = .0006**; ES = 0.12	1.8 (–5.9 to 35.1) *P* > .99; ES = 0.07
Collateral ligament injury					
MCL	34 (0.2)	11 (1.4)	1 (7.7)	−2.2 (–3.3 to −1.1) ***P* = .0015**; ES = 0.13	5.7 (–1.7 to 39) *P* = .48; ES = 0.27
LCL	171 (0.9)	9 (1.2)	1 (7.7)	0.3 (–0.5 to 1.2) *P* = .45; ES = 0.03	6.5 (–0.9 to 40) *P* = .31; ES = 0.32
PLC injury	34 (0.2)	11 (1.4)	1 (7.7)	1.3 (0.4 to 2.2) ***P* < .0001**; ES = 0.14	6.2 (–1.2 to 40) *P* = .37; ES = 0.30
Meniscus injury and treatment					
Lateral meniscus injury	4874 (24.7)	150 (19.7)	1 (7.7)	−4.9 (–7.9 to −2) ***P* = .0018**; ES = 0.12	−12 (–20.4 to 22.3) *P* = .49; ES = 0.36
Lateral meniscus treatment				***P* = .0016**	*P* = .91
Lateral meniscus repair	641 (3.2)	35 (4.6)	0 (0.0)	1.4 (–0.2 to 2.9) *P* = .059; ES = 0.07	−4.6 (–7.2 to 27.4) *P* > .99; ES = 0.31
Lateral meniscus resection	3,346 (16.9)	98 (12.9)	1 (7.7)	−4.0 (–6.5 to −1.5) ***P* = .0032**; ES = 0.11	−5.2 (–13.2 to 28.5) *P* = .98; ES = 0.17
Lateral meniscus repair + resection	53 (0.3)	1 (0.1)	0 (0)	−0.1 (–0.5 to 0.2) *P* = .80; ES = 0.03	−0.1 (–1.6 to 31.6) *P* > .99; ES = 0.05
Lateral meniscus injury left in situ	834 (4.2)	16 (2.1)	0 (0)	−2.1 (–3.2 to −1) ***P* = .0026**; ES = 0.12	−2.1 (–4.1 to 29.8) *P* > .99; ES = 0.21
Medial meniscus injury	5,196 (26.3)	170 (22.4)	2 (15.4)	−3.9 (–7.0 to −0.8) ***P* = .016**; ES = 0.09	−7.0 (–20.1 to 27.7) *P* = .84; ES = 0.18
Medial meniscus treatment				*P* = .095	*P* = .30
Medial meniscus repair	1,167 (5.9)	50 (6.6)	0 (0.0)	0.7 (–1.2 to 2.5) *P* = .48; ES = 0.03	−6.6 (–9.4 to −25.9) *P* = .83; ES = 0.38
Medial meniscus resection	3,284 (16.6)	101 (13.3)	1 (7.7)	−3.3 (–5.9 to −0.8) ***P* = .015**; ES = 0.09	−5.6 (–13.6 to 28.5) *P* = .94; ES = 0.18
Medial meniscus repair + resection	32 (0.2)	2 (0.3)	0 (0.0)	0.1 (–0.3 to 0.5) *P* = .72; ES = 0.02	−0.3 (–1.7 to 31.7) *P* > .99; ES = 0.07
Medial meniscus injury left in situ	713 (3.6)	17 (2.2)	1 (7.7)	−1.4 (–2.5 to −0.2) ***P* = .046**; ES = 0.08	5.5 (–2.0 to 38.9) *P* = .53; ES = 0.25
ACL graft type				***P* < .0001**	***P* < .0001**
Patellar tendon autograft	977 (5)	489 (65.1)	6 (50.0)	60.1 (56.6 to 63.6) ***P* < .0001**; ES = 1.62	−15.1 (–44.6 to 15.3) *P* = .43; ES = 0.31
Semitendinosus autograft	18,129 (93)	152 (20.2)	0 (0.0)	−72.7 (–75.7 to 69.8) ***P* < .0001**; ES = 2.16	−20.2 (–24.2 to 15.1) *P* = .14; ES = 0.71
Quadriceps tendon autograft	300 (1.5)	65 (8.7)	1 (8.3)	7.1 (85 to 9.2) ***P* < .0001;** ES = 0.33	−0.3 (–8.7 to 35.2) *P* > .99; ES = 0.01
Allograft	56 (0.3)	38 (5.1)	5 (41.7)	4.8 (3.1 to 6.4) ***P* < .0001**; ES = 0.30	36.6 (9.8 to 68.7) ***P* = .0005**; ES = 0.96
Direct suture/synthetic/other	39 (0.2)	7 (0.9)	0 (0.0)	0.7 (–0.0 to 1.5) ***P* = .0028**; ES = 0.10	−0.9 (–2.7 to 33.2) *P* = 1.00; ES = 0.14
Time from injury to surgery, mo	20.5 ± 37.0 8.2 (0-551)	14.5 ± 17.2 7.4 (1.8-17.1)	28.6 ± 32.6 22.1 (2.9-112.9)	6.1 (3.2 to 8.8) ***P* = .0020**; ES = 0.166	−14.1 (–21.9 to −2) ***P* = .022**; ES = 0.807
Time from ACLR to most recent revision, y	—	2.8 ± 2.1 2.1 (0-13.8)	5.3 ± 2.9 23.6 (21.2-29.4)	—	—
Follow-up after most recent surgery, y	9.3 ± 4.0 9.3 (2.2-17.16)	7.7 ± 3.4 7.4 (1.8-17.1)	6.1 ± 2.6 5.4 (2.5-11.3)	1.7 (1.4 to 2) ***P* = .0020**; ES = 0.422	1.60 (–0.3 to 3.5) *P* = .078; ES = 0.474

a Data are reported as n (%) for categorical variables and mean ± SD and median and minimum and maximum for continuous variables unless otherwise indicated. The sums may vary because of missing values—variables with missing values were BMI, time from injury to surgery, meniscus treatment, cartilage injury, ACL graft type, and activity at the time of injury. Bold *P* values indicate a statistically significant difference between groups compared (*P* < .05). ACL, anterior cruciate ligament; ACLR, ACL reconstruction; BMI, body mass index; ES, effect size; LCL, lateral collateral ligament; MCL, medial collateral ligament; MD, mean difference; mr-ACLR, multiple-revision ACLR; PLC, posterior lateral corner; r-ACLR, revision ACLR.

b Pivoting sport—American football/rugby, basketball, dancing, floorball, gymnastics, handball, ice hockey/bandy, martial arts, racket sports, soccer, volleyball, wrestling; nonpivoting sport—cross-country skiing, cycling, horseback riding, motocross/endure, skateboarding, snowboarding, and surfing/wakeboarding; other physical activity—other recreational sports, exercise, trampoline; and other—other outdoor activity and work.

The age at the time of the most recent ACLR was found to be significantly different between the groups, with patients in the r-ACLR group being significantly younger than patients in the primary ACLR group (25.8 vs 29.1 years; *P* = .0020) (Table 1). In addition, patients in the primary ACLR group were found to have significantly fewer concomitant cartilage injuries in the lateral femoral condyle (5.7% vs 9.5%; *P* < .0001), the medial femoral condyle (18.6% vs 25.8%; *P* < .0001), the lateral tibial plateau (6.9% vs 9.2%; *P* = .020), the medial tibial plateau (5.4% vs 7.4%; *P* = .025), and the trochlea (3.4% vs 5.9%; *P* = .0006) compared with patients undergoing r-ACLR. No significant difference was found in the amount of concomitant cartilage injuries between patients undergoing r-ACLR and mr-ACLR. The primary ACLR group had a significantly greater proportion of concomitant lateral (24.7% vs 19.7%; *P* = .0018) and medial meniscus injuries (26.3% vs 22.4%; *P* = .016) compared with the r-ACLR group. Graft choices were significantly different among the groups, with allografts being the most common treatment options in the mr-ACLR group and semitendinosus autografts and patellar tendon autografts being the most common graft choices in the primary ACLR and r-ACLR groups, respectively (Table 1).

### Pre- and Postoperative KOOS

Preoperatively, patients who underwent r-ACLR had significantly higher preoperative KOOS-Sport/Rec scores than patients who underwent primary ACLR (42 vs 39.7, respectively; *P* = .027). In addition, patients undergoing r-ACLR had significantly higher preoperative KOOS-Pain (75.8 vs 74.3; *P* = .023) and KOOS-ADL (87.3 vs 83.1; *P* < .0001) compared with patients who had primary ACLR (Table 2). However, no significant differences were found in preoperative KOOS subscores between the patients in the r-ACLR and mr-ACLR groups (Table 3).

**Table 2:** Pre- and Postoperative KOOS and Changes in the KOOS Between the Primary ACLR and r-ACLR Groups^*a*^

Variable	Primary ACLR		r-ACLR		Primary ACLR vs r-ACLR			
	Value	Adj Mean^*b*^ SEM	Value	Adj Mean^*b*^ SEM	Adj MD (95% CI)	*P*	Adj *P*	ES
Preoperative								
KOOS-Pain	74.3 ± 17.7; 77.8 (0 to 100); n = 19,759	—	75.8 ± 18.3; 80.6 (13.9 to 100); n = 760	—	—	**.023**	—	0.084
KOOS-Symptoms	69.5 ± 18.6; 71.4 (0 to 100); n = 19,763	—	70.6 ± 18.7; 71.4 (10.7 to 100); n = 760	—	—	.097	—	0.061
KOOS-Sport/Rec	39.7 ± 27.4; 35 (0 to 100); n = 19,732	—	42.0 ± 28.1; 40 (0 to 100); n = 760	—	—	**.027**	—	0.082
KOOS-QoL	33 ± 18.6; 31.3 (0 to 100); n = 19,744	—	32.9 ± 21.3; 31.3 (0 to 100); n = 760	—	—	.34	—	0.040
KOOS-ADL	83.1 ± 17.3; 88.2 (0 to 100); n = 19,757	91.5; 0.1 (91.4 to 91.7)	87.3 ± 16.2; 94.1 (2.9 to 100); n = 759	87.9; 0.5 (87 to 88.9)	3.59 (2.6 to 4.6)	**<.0001**	**<.0001**	0.334
KOOS-4	54.3 ± 17.5; 54.4 (1.3 to 100); n = 19,708	—	55.3 ± 18.8; 54.9 (8.8 to 100); n = 760	—	—	.14	—	0.059
Postoperative								
KOOS-Pain	84.6 ± 15.2; 88.9 (0 to 100); n = 19,759	84.5; 0.1 (84.3 to 84.7)	78.2 ± 18.1; 83.3 (8.3 to 100); n = 760	79.8; 0.6 (78.6 to 80.9)	4.74 (3.6 to 5.9)	**<.0001**	**<.0001**	0.416
KOOS-Symptoms	77.0 ± 17.6; 78.6 (0 to 100); n = 19,763	76.9; 0.1 (76.7 to 77.2)	70.7 ± 19.6; 71.4 (0 to 100); n = 760	72.7; 0.7 (70.8 to 73.6)	4.75 (3.4 to 6.1)	**<.0001**	**<.0001**	0.355
KOOS-Sport/Rec	64.8 ± 26.5; 70 (0 to 100); n = 19,732	64.5; 0.2 (64.2 to 64.9)	51.0 ± 28.1; 55 (0 to 100); n = 760	55.5; 1 (53.3 to 57.5)	8.99 (6.9 to 11.1)	**<.0001**	**<.0001**	0.517
KOOS-QoL	59.1 ± 22.9; 62.5 (0 to 100); n = 19,744	58.9; 0.2 (58.6 to 59.2)	44.5 ± 23.3; 43.8 (0 to 100); n = 760	48; 0.9 (46.2 to 49.8)	10.9 (9.1-12.7)	**<.0001**	**<.0001**	0.633
KOOS-ADL	91.6 ± 12.8; 97.1 (0 to 100); n = 19,757	91.5; 0.1 (91.4 to 91.7)	87.3 ± 16.2; 94.1 (2.9 to 100); n = 759	87.9; 0.5 (87.0 to 88.9)	3.59 (2.6 to 4.6)	**<.0001**	**<.0001**	0.334
KOOS-4	71.4 ± 18.5; 74.4 (0 to 100); n = 19,708	71.2; 0.1 (71 to 71.5)	61.1 ± 20.0; 62.7 (3.7 to 100); n = 760	63.8; 0.7 (62.4 to 65.2)	7.40 (6.0 to 8.8)	**<.0001**	<**.0001**	0.552
Δ_preop-postop_								
KOOS-Pain	10.3 ± 17.8; 8.3 (–80.6 to 91.7); n = 19,759	10.3; 0.1 (10.1 to 10.4)	2.5 ± 18.0; 0 (–58.3 to 72.2); n = 760	5.5; 0.6 (4.4 to 6.7)	4.7 (3.6 to 5.9)	**<.0001**	**<.0001**	0.442
KOOS-Symptoms	7.6 ± 20.6; 7.1 (–82.1 to 92.9); n = 19,763	7.5; 0.12 (7.3 to 7.8)	0.1 ± 19.2; 0 (–64.3 to 64.3); n = 760	2.8; 0.70 (1.4 to 4.14)	4.8 (3.4 to 6.1)	**<.0001**	**<.0001**	0.361
KOOS-Sport/Rec	25.0 ± 29.9; 25 (–100 to 100); n = 19,732	24.9; 0.2 (24.5 to 25.2)	9.0 ± 29.6; 7.5 (–95 to 100); n = 760	15.9; 1.0 (13.9 to 17.9)	9.0 (6.9 to 11.1)	**<.0001**	**<.0001**	0.535
KOOS-QoL	25.4 ± 24.6; 25 (–100 to 100); n = 19,744	25.3; 0.2 (25 to 25.7)	11.7 ± 24.0 12.5 (–75 to 93.8); n = 760	14.4; 0.9 (12.7 to 16.2)	10.9 (9.1 to 12.7)	**<.0001**	**<.0001**	0.559
KOOS-ADL	8.5 ± 16.4; 4.41 (–98.53 to 100); n = 19,757	8.5; 0.08 (8.3 to 8.6)	2.3 ± 15.4; 0 (–54.4 to 82.4); n = 759	4.9 0.5 (3.9 to 5.8)	3.6 (2.6 to 4.6)	**<.0001**	**<.0001**	0.378
KOOS-4	17.1 ± 19.5; 17.2 (–74.3 to 93.5); n = 19,708	17.0; 0.1 (16.8 to 17.2)	5.8 ± 19.1; 4.8 (–63.8 to 71.4); n = 760	9.6 0.7 (8.2 to 11.0)	7.4 (6.0 to 8.2)	**<.0001**	**<.0001**	0.578

a Data are reported as mean ± SD and median and minimum and maximum unless otherwise indicated. The quantity of follow-up data may vary among the KOOS subgroups; thus, the numbers within the different KOOS subgroups encompass the count of patients who completed their respective KOOS subgroup questionnaire. Dashes indicate areas not applicable. Bold *P* values indicate a statistically significant difference between groups (*P* < .05). ACLR, anterior cruciate ligament reconstruction; Adj, adjusted; ADL, activities of daily living; ES, effect size; KOOS, Knee injury and Osteoarthritis Outcome Score; MD, mean difference; postop, postoperative; preop, preoperative; QoL, quality of life; r-ACLR, revision ACLR; Sport/Rec, Function in Sport and Recreation.

b Adjusted for respective preoperative scores and baseline characteristics by using analysis of covariance.

**Table 3:** Pre- and Postoperative KOOS Scores and Changes in the KOOS Scores Between the r-ACLR and mr-ACLR Groups^*a*^

Variable	r-ACLR		mr-ACLR		r-ACLR vs mr-ACLR			
	Value	Adj Mean^*b*^ SEM	Value	Adj Mean^*b*^ SEM	Adj MD (95% CI)	*P*	Adj *P*	ES
Preoperative								
KOOS-Pain	75.8 ± 18.3; 80.6 (13.9 to 100); n = 760	—	68.2 ± 30.4; 80.6 (5.6 to 100); n = 13	—	—	.38	—	0.410
KOOS-Symptoms	70.6 ± 18.7; 71.4 (10.7 to 100); n = 760	—	70.6 ± 18.7; 1.4 (10.7 to 100); n = 13	62.4 ± 22.1; 67.9 (21.4 to 92.9)	—	—	.12	0.440
KOOS-Sport/Rec	42.0 ± 28.1; 40 (0 to 100); n = 760	—	40.4 ± 34.1; 40 (0 to 100); n = 13	—	—	.84	—	0.056
KOOS-QoL	32.9 ± 21.3; 31.3 (0 to 100); n = 760	—	30.8 ± 23.6; 31.3 (0 to 75); n = 13	—	—	.72	—	0.100
KOOS-ADL	87.3 ± 16.2; 94.1 (2.9 to 100); n = 759	87.9; 0.5 (87 to 88.9)	74.8 ± 30.8; 91.2 (23.5 to 100); n = 13	—	—	.26	—	0.590
KOOS-4	55.3 ± 18.8; 54.9 (8.8 to 100); n = 760	—	50.4 ± 25.1; 54.7 (11.2 to 82.8); n = 13	—	—	.36	—	0.258
Postoperative								
KOOS-Pain	78.2 ± 18.1; 83.3 (8.3 to 100); n = 760	78.2; 0.6 (77.0 to 79.3)	59.2 ± 33.2; 66.7 (5.6 to 94.4); n = 13	63.2; 4.4 (54.6 to 71.8)	15.0 (6.3 to 23.6)	.061	**.0007**	1.03
KOOS-Symptoms	70.7 ± 19.6; 71.4 (0 to 100); n = 760	70.7; 0.6 (69.5 to 71.9)	58.8 ± 28.4; 57.1 (17.9 to 100); n = 13	63.0; 4.8 (53.7 to 72.4)	7.6 (–1.8 to 17.1)	.16	.11	0.606
KOOS-Sport/Rec	51.0 ± 28.1; 55 (0 to 100); n = 760	51.0; 0.9 (49.2 to 52.8)	40.8 ± 36.9; 40 (0 to 95); n = 13	41.8; 7.0 (28 to 55.5)	9.2 (–4.7 to 23.1)	.20	.19	0.362
KOOS-QoL	44.5 ± 23.3; 43.8 (0 to 100); n = 760	44.5; 0.8 (43 to 46)	39.4 ± 28.5; 37.5 (0 to 81.3); n = 13	40.3; 5.9 (28.7-51.8)	4.3 (–7.4 to 15.9)	.43	.47	0.219
KOOS-ADL	87.3 ± 16.2; 94.1 (2.9 to 100); n = 759	87.2; 0.5 (86.2 to 88.1)	73.0 ± 28.6; 88.2 (22.1 to 100); n = 13	78.6; 3.7 (71.3 to 85.9)	8.5 (1.2 to 15.9)	.097	**.023**	0.868
KOOS-4	61.1 ± 20.0; 62.7 (3.7 to 100); n = 760	61.1 0.6 (59.9 to 62.3)	49.5 ± 30.8; 50.9 (5.9 to 87.5); n = 13	52.2 4.8 (42.8 to 61.6)	8.9 (–0.6 to 18.4)	.20	.067	0.573
Δ_preop-postop_								
KOOS-Pain	2.5 ± 18.0; 0 (–58.3 to 72.2) n = 760	2.5; 0.6 (1.4 to 3.6)	−8.97 ± 23.3 –5.6 (–80.6 to 8.3); n = 13	−12.5; 4.4 (–21.1 to −3.9)	15.0 (6.3 to 23.6)	**.024**	**.0007**	0.631
KOOS-Symptoms	0.1 ± 19.2; 0 (–64.3 to 64.3); n = 760	0.2 0.6 (–1 to 1.4)	−3.6 ± 19.9; 0 (–53.6 to 21.4); n = 13	−7.4 4.8 (–16.8 to 1.9)	7.6 (–1.8 to 17.1)	.49	.11	0.193
KOOS-Sport/Rec	9.0 ± 29.6; 7.5 (–95 to 100); n = 760	9.1; 0.9 (7.3 to 10.9)	0.39 ± 35.7; 0 (–100 to 45); n = 13	−0.2; 7 (–14.0 to 13.6)	9.2 (–4.7 to 23.1)	.30	.19	0.291
KOOS-QoL	11.7 ± 24.0; 12.5 (–75 to 93.8) n = 760	11.7 0.8 (10.2 to 13.2)	8.7 ± 26.8; 6.3 (–37.5 to 50)	7.4 5.9 (–4.2 to 19)	4.3 (–7.4 to 15.9)	.66	.47	0.125
KOOS-ADL	2.3 ± 15.34; 0 (–54.41 to 82.35) n = 759	2.4 0.5 (1.4 to 3.3)	−1.8 ± 16.5; 0 (–51.5 to 23.5); n = 13	−6.16 3.71 (–13.45 to 1.14)	8.5 (1.2 to 15.9)	.34	**.023**	0.267
KOOS-4	5.8 ± 19.1 4.84 (–63.8 to 71.4); n = 760	5.9 0.6 (4.6 to 7.1	−0.9 ± 23.9; –0.3 (–67.9 to 27.7) n = 13	−30.2 4.80 (–12.44 to 6.41)	8.9 (–0.6 to 18.4)	.21	.067	0.348

a Data are reported as mean ± SD and median and minimum and maximum unless otherwise indicated. The quantity of follow-up data may vary among the KOOS subgroups; thus, the numbers within the different KOOS subgroups encompass the count of patients who completed their respective KOOS subgroup questionnaire. Dashes indicate areas not applicable. Bold *P* values indicate a statistically significant difference between groups (*P* < .05). ACLR, anterior cruciate ligament reconstruction; Adj, adjusted; ADL, activities of daily living; ES, effect size; KOOS, Knee injury and Osteoarthritis Outcome Score; MD, mean difference; mr-ACLR, multiple-revision ACLR; postop, postoperative; preop, preoperative; QoL, quality of life; r-ACLR, revision ACLR; Sport/Rec, Function in Sport and Recreation.

b Adjusted for respective preoperative score and age at surgery using analysis of covariance.

**Figure 2.**
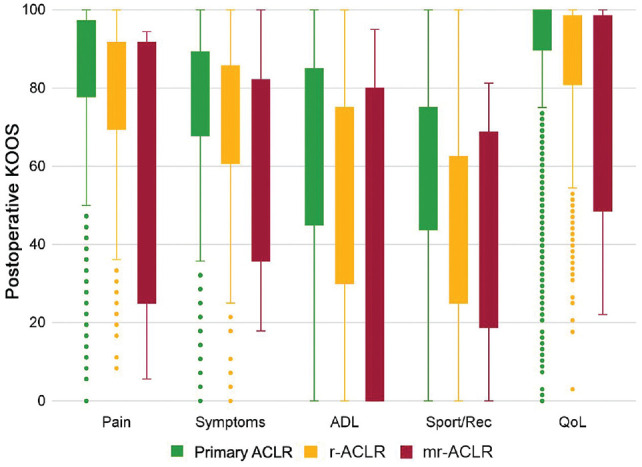
Boxplot illustrating the distribution of the postoperative KOOS across the primary ACLR, r-ACLR, and mr-ACLR groups. The boxes indicate first and third quartiles as well as median values. Error bars indicate minimum and maximum values, and dots indicate outliers. ACLR, anterior cruciate ligament reconstruction; ADL, activities of daily living; KOOS, Knee injury and Osteoarthritis Outcome Score; mr-ACLR, multiple-revision ACLR; QoL, quality of life; r-ACLR, revision ACLR; Sport/Rec, Function in Sport and Recreation.

All postoperative KOOS subscores were significantly different between the primary ACLR and r-ACLR groups (adjusted *P* [*P*_adj_] < .0001 for all) (Table 2 and Figure 2). Furthermore, patients undergoing mr-ACLR had significantly lower adjusted postoperative KOOS-Pain (63.2 vs 78.2; *P*_adj_ = .0007) and KOOS-ADL (78.6 vs 87.2; *P*_adj_ = .023) compared with those undergoing r-ACLR (Table 3).

### Changes Between Pre- and Postoperative KOOS

Significant differences were found in changes from pre- to postoperative KOOS scores between patients undergoing primary ACLR and r-ACLR (Table 2). Patients in the r-ACLR group were found to have significantly smaller improvements on all KOOS subscales compared with patients in the primary ACLR group (*P*_adj_ < .0001 for all) (Table 2). Moreover, a significantly smaller improvement was observed in KOOS-Pain after mr-ACLR compared with r-ACLR (–9.0 ± 23.3 vs 2.5 ± 18.0, respectively; *P* = .024) (Table 3).

## Discussion

The key findings of this study indicate that primary ACLR results in the most significant improvement in the KOOS subscales, with a subsequent decrease in improvement after 1 revision and minimal improvement after multiple revisions. Moreover, only primary ACLR had improvements between pre- and postoperative KOOS subscales that reached the minimum important change (MIC)^22^ for all the 5 subscales. These findings suggest that mr-ACLR may lead to more inferior knee-related outcomes.

A significant difference in postoperative KOOS was found between the patients undergoing primary ACLR, r-ACLR, and mr-ACLR, which is consistent with previous literature reporting poorer postoperative outcomes associated with r-ACLR.^23,25,38^ For instance, patients who had r-ACLR were found to have lower postoperative KOOS-Sport/Rec (51.0 vs 64.8) and KOOS-QoL (44.5 vs 59.1) compared with patients who underwent primary ACLR. Also, patients who underwent mr-ACLR exhibited lower postoperative scores for the KOOS subscales, such as Pain, compared with the r-ACLR group (63.2 vs 78.2). Several factors may have contributed to the poorer outcomes observed among patients undergoing r-ACLR and mr-ACLR. Specifically, patients in these groups were younger at the time of their primary ACLR than patients requiring only 1 ACLR. Younger patients may have more exposure to high-risk activity and strenuous demands upon returning to activity after ACLR, placing them at increased risk of failure. In addition, research has shown that younger patients may have unrealistic expectations for their functional knee recovery after surgery, which has been shown to influence patient-reported outcomes negatively.^16^ Furthermore, correction of sagittal and coronal malalignment or addressing concomitant soft tissue injuries in the setting of mr-ACLR may have contributed to the poorer postoperative outcomes.^9,18,25,31,35^ However, our dataset did not include information on these concomitant procedures. This study showed almost double the incidence of cartilage injuries in patients undergoing mr-ACLR compared with patients undergoing primary ACLR. Interestingly, the incidence of medial meniscus injuries was approximately the same between the 3 groups. Thus, a higher incidence of concomitant cartilage injuries may have impacted the outcomes after mr-ACLR.^29,36^ Also, the psychological status may play a role in postoperative outcomes, as data have shown that emotional barriers can have a negative impact on surgical outcomes,^6,25^ particularly in cases involving multiple surgeries followed by extended rehabilitation, which has the potential to negatively affect a patient's psychological state.^21^ Last, the high degree of allograft use (42%) in patients undergoing mr-ACLR with a mean age of 28 years may have subsequently contributed to the inferior postoperative outcomes. A patellar tendon or quadriceps tendon autograft may have been a more suitable option, as a recent systematic review has shown that even allograft use in the revision setting may result in inferior outcomes compared with autograft use.^4^ In addition, other factors may have contributed to the poorer outcomes observed after r-ACLR and mr-ACLR, including increased quadriceps atrophy resulting from multiple procedures, as reduced quadriceps strength after ACL injury and subsequent construction has previously been reported.^10,13^

When examining the comparisons with statistical significance in the KOOS Pain and ADL subscales, differences in mean values between the r-ACLR and mr-ACLR groups were higher than the previously reported minimal clinically important difference (MCID) for the respective KOOS subscale score (19.0 vs 11.9 and 14.3 vs 13.3).^3^ These findings suggest that the difference in postoperative outcomes between r-ACLR and mr-ACLR are not only statistically significant but clinically meaningful and imply that patients undergoing r-ACLR experience a more notable improvement in pain and ADL compared with those undergoing mr-ACLR.

In this study, the most significant improvement in patient-reported knee function occurred after primary ACLR, with a subsequent decrease in improvement after a single revision and minimal improvement after multiple revisions. Thus, only patients with primary ACLR achieved an MIC for all 5 KOOS subscales.^22^ The MIC values were previously described as 12.1 for the KOOS Sport/Rec, 18.3 for the KOOS QoL, –1.2 for the KOOS Symptoms, 2.5 for the KOOS ADL, and 2.5 for the KOOS Pain.^22^ Most importantly, patients undergoing primary ALCR achieved the MIC for the KOOS Sport/Rec and QoL subscales, which are most reliable when assessing outcomes after ACLR.^22^ However, the results of this study still show that patients undergoing r-ACLR experienced an improvement in every KOOS subscale despite not achieving an MIC. This result suggests that r-ACLR can still lead to some improvement, although it may not be as clinically significant as primary ACLR. However, the established MIC values for primary ACLR may not apply to revision cases. Using them to evaluate clinical improvement in patients undergoing r-ACLR and mr-ACLR may lead to somewhat misleading results. Previous research has demonstrated that patients with mr-ACLR can return to sports, and patients who underwent mr-ACLR had significantly better functional outcome scores, such as the Tegner activity score and the KOOS Sport/Rec and QoL, compared with those treated nonoperatively.^17^ Despite the common occurrence of r-ACLR during the study period, only 13 patients with preoperative and 1-year outcome data underwent mr-ACLR, notably fewer than numbers reported by single-center studies conducted in other countries.^15,17,18^ However, this difference could be due to patients’ preferences to not undergo surgery and changing lifestyles. These findings suggest that surgical treatment for revision ACLs may not be necessary in all cases, highlighting the importance of acknowledging patients’ functional goals when making a shared decision to undergo a second r-ACLR.^34^

The results of this study have important clinical implications, as they provide valuable information on the differences between pre- and postoperative outcomes in patients undergoing primary ACLR, r-ACLR, and mr-ACLR. The study findings suggest that only primary ACLR results in clinically meaningful improvement in the KOOS from pre- to postoperative time points. While postoperative functional outcomes in this study were poorer for r-ACLR and mr-ACLR, patients undergoing r-ACLR or mr-ACLR surgeries also showed improvement after surgery. Nevertheless, the improvement was less compared with the primary ACLR group. Thus, it is important to strive for the best possible outcome in primary ACLR, utilizing a safe and proper approach to recovery and minimizing the risk for failure and the need for revision of ACLR. Surgeons should consider these findings when selecting and managing patients for r-ACLR, particularly mr-ACLR, and strive to improve patient awareness of the expected outcomes. Preoperative education may include counseling patients about the potential limitations of revision surgeries and setting realistic expectations for postoperative outcomes.

### Limitations

This study has limitations that should be acknowledged. Although the sample size was large, using registry data may limit the ability to draw definitive conclusions about cause-and-effect relationships. Considering that the primary aim of the SNKLR is to collect data on surgically treated primary ACL injuries, it is possible that some revision cases might have been missed, and the small number of included mr-ACLR cases can partly be attributed to missing pre- and/or postoperative outcome data. Moreover, the KOOS was validated for patients with knee osteoarthritis; therefore, the construct validity for assessing outcomes after ACLR may be limited.^8,20^ In addition, it may not fully capture all the functional limitations associated with ACL surgery, as other factors, such as patient expectations and psychological status, may also affect outcomes.^7,26,33^ It is also possible that the assessment of 1-year outcomes, especially for revision cases, was too early, which could have subsequently influenced the results. In addition, the MIC and MCID values discussed in this study were based on mean values and not calculated separately for each patient. Furthermore, the MIC and MCID values have primarily been determined for primary ACLR and may not be as accurate when looking at revision cases. Therefore, future studies may benefit from using additional assessment tools and establishing MIC and MCID cutoffs for revision cases to comprehensively evaluate the functional outcomes in patients undergoing ACLR, including those undergoing revision and multiple revision surgeries. Finally, the absence of logistic regression analyses may have introduced bias, as some confounding variables affecting the outcome scores may have been overlooked.

## Conclusion

The results of this study indicated an improvement in knee function after primary ACLR, r-ACLR, and mr-ACLR. The most notable improvement in functional outcomes occurred after primary ACLR. Furthermore, lower postoperative outcome scores were found in patients undergoing at least 1 r-ACLR, and even worse outcomes were seen for mr-ACLR. Primary ACLR may provide the best chance for recovery after ACL injury. It is important to strive for the best possible outcome in primary ACLR, minimizing the need for revision. Surgeons should consider these study findings when counseling patients undergoing primary and r-ACLR surgery.

## Supplemental Material

sj-pdf-1-ojs-10.1177_23259671231217725 – Supplemental material for Comparison of Improvement in Patient-Reported Knee Function After Revision and Multiple-Revision ACL Reconstruction Compared With Primary ACL Reconstruction Click here for additional data file. Supplemental material, sj-pdf-1-ojs-10.1177_23259671231217725 for Comparison of Improvement in Patient-Reported Knee Function After Revision and Multiple-Revision ACL Reconstruction Compared With Primary ACL Reconstruction by Janina Kaarre, Zachary J. Herman, Alberto Grassi, Eric Hamrin Senorski, Volker Musahl and Kristian Samuelsson in Orthopaedic Journal of Sports Medicine
