# Supplementary material for: Comparison of Improvement in Patient-Reported Knee Function After Revision and Multiple-Revision ACL Reconstruction Compared With Primary ACL Reconstruction
Source: Orthop J Sports Med. 2023 Dec 22;11(12):23259671231217725. doi: 10.1177/23259671231217725 (PMC10748942; doi:10.1177/23259671231217725)
Supplement: sj-pdf-1-ojs-10.1177_23259671231217725 – Supplemental material for Comparison of Improvement in Patient-Reported Knee Function After Revision and Multiple-Revision ACL Reconstruction Compared With Primary ACL Reconstruction [file sj-pdf-1-ojs-10.1177_23259671231217725.pdf]

Supplemental Table S1. Baseline Characteristics of the Included Patients vs Patients Who Were Excluded Due to Missing KOOS Data

Variable	KOOS preop and/or KOOS postop data missing (n=8,520)	Included patients (n=20,542)	*P*<br>MD (95% CI)<br>ES
**Age at surgery (y)**	28.4 ± 10.4<br>26 (16-72)	28.8 ± 10.6<br>26 (16-74)	
**Sex (male)**	4,750 (55.8)	10,837 (52.8)	**<0.0001**<br>-3 (-4.3 to 0-1.7)<br>0.06
**BMI (kg/m²)**	24.9 ± 3.4<br>14.4 (16.9-49.8)	24.7 ± 3.3<br>24.2 (15.1-47.1)	
**Activity during injury**			**<0.0001**
Alpine/skiing	1,219 (14.3)	3,593 (17.3)	**<0.0001**<br>2.9 (2.0 to 3.8)<br>0.08
Pivoting sport	5,416 (63.7)	13,103 (63.9)	0.74<br>0.2 (-1.0 to 1.4)<br>0.00
Non-pivoting sport	395 (4.6)	882 (4.3)	0.21<br>-0.3 (-0.9 to 0.2)<br>0.02
Other physical activity	397 (4.7)	817 (4.0)	**0.0095**<br>-0.7 (-1.2 to -0.2)<br>0.03
Traffic	186 (2.2)	338 (1.6)	**0.0024**<br>-0.5 (-0.9 to -0.2)<br>0.04
Other	888 (10.4)	1,818 (8.9)	**<0.0001**<br>-1.6 (-2.3 to -0.8)<br>0.05
**Cartilage injury**			
Lateral femoral condyle	474 (5.6)	1,150 (5.6)	0.93<br>0.0 (-0.6 to 0.6)<br>0.00
Medial femoral condyle	1,629 (19.1)	3,797 (18.5)	0.21<br>-0.6 (-1.6 to 0.4)<br>0.02
Lateral patella	205 (2.4)	679 (3.3)	**<0.0001**<br>0.9 (0.5 to 1.3)<br>0.05
Medial patella	422 (5.0)	1,073 (5.2)	0.36<br>0.3 (-0.3 to 0.8)<br>0.01
Lateral tibial plateau	509 (6.0)	1,394 (6.8)	**0.011**<br>0.8 (0.2 to 1.4)<br>0.03
Medial tibial plateau	468 (5.5)	1,081 (5.3)	0.44<br>-0.2 (-0.8 to 0.3)<br>0.01
Trochlea	229 (2.7)	675 (3.3)	**0.0076**<br>0.6 (0.2 to 1.0)<br>0.04
**Collateral ligament injury**			
MCL	404 (4.7)	865 (4.2)	**0.048**<br>-0.5 (-1.1 to 0.0)<br>0.03
LCL	125 (1.5)	177 (0.9)	**<0.0001**<br>-0.6 (-0.9 to -0.3)<br>0.06
**PLC injury**	31 (0.4)	35 (0.2)	**0.0036**<br>-0.2 (-0.3 to 0.0)<br>0.04
**Meniscus injury and treatment**			
Lateral meniscus injury	1,938 (22.7)	5,090 (25.8)	**0.0002**<br>2.0 (1.0 to 3.1)<br>0.05

Variable	KOOS preop and/or KOOS postop data missing (n=8,520)	Included patients (n=20,542)	*P*<br>MD (95% CI)<br>ES
Lateral meniscus treatment			0.32
Lateral meniscus repair	252 (13.0)	664 (13.0)	0.24<br>0.3 (-0.2 to 0.7)<br>0.02
Lateral meniscus resection	1,295 (66.8)	3,493 (68.6)	**0.0002**<br>1.8 (0.9 to 2.7)<br>0.05
Lateral meniscus repair + resection	26 (1.3)	55 (1.1)	0.66<br>-0.0 (-0.2 to 0.1)<br>0.01
Lateral meniscus injury left in situ	365 (18.8)	878 (17.2)	0.99<br>-0.0 (-0.5 to 0.5)<br>0.0
Medial meniscus injury	2,265 (26.6)	5,364 (26.1)	0.41<br>-0.5 (-1.6 to 0.7)<br>0.01
Medial meniscus treatment			**0.0022**
Medial meniscus repair	425 (18.8)	1,200 (22.4)	**0.0040**<br>0.9 (0.3 to 1.4)<br>0.04
Medial meniscus resection	1,474 (65.1)	3,395 (63.3)	0.11<br>-0.8 (-1.7 to 0.2)<br>0.02
Medial meniscus repair + resection	19 (0.8)	33 (0.6)	0.32<br>-0.1 (-0.2 to 0.1)<br>0.01
Medial meniscus injury left in situ	347 (15.3)	736 (13.7)	**0.049**<br>-0.5 (-1.0 to 0.0)<br>0.03
**ACL graft type**			**<0.0001**
Patellar tendon autograft	678 (8.0)	1,011 (5.0)	**<0.0001**<br>-3.0 (-3.7 to -2.4)<br>0.12
Semitendinosus autograft	7,594 (89.7)	18,839 (93.0)	**<0.0001**<br>3.2 (2.5 to 4.0)<br>0.12
Quadriceps tendon autograft	136 (1.6)	311 (1.5)	0.69<br>-0.1 (-0.4 to 0.3)<br>0.01
Allograft	37 (0.4)	60 (0.3)	0.082<br>-0.1 (-0.3 to 0.0)<br>0.02
Direct suture/synthetic/other	18 (0.2)	40 (0.2)	0.89<br>-0.0 (-0.1 to 0.1)<br>0.00
**Time from injury to surgery (mo)**	21.0 ± 34.8<br>9.2 (0-389.3)	20.2 ± 36.6<br>8 (0-551)	0.090<br>0.804 (-0.218 to 1.738)<br>0.022
**Time from index ACLR to most recent revision (y)**	0.342 ± 1.4<br>0 (0-14.4)	0.107 ± 0.7<br>0 (0-13.8)	
**Follow-up after most recent surgery (y)**	21.0 ± 34.8<br>9.2 (0-389.3)	20.2 ± 36.6<br>8 (0-551)	0.090<br>—<br>—
**Study group**			**<0.0001**<br>—<br>—
Primary ACLR	7781 (91.3)	19,769 (96.2)	
Single revision ACLR	709 (8.3)	760 (3.7)	
Multiple revision ACLR	30 (0.4)	13 (0.1)	

Data are reported as n (%) for categorical variables and as mean ± SD and median as well as minimum and maximum for continuous variables. The sums may vary because of missing values (variables with missing values were BMI, time from injury to surgery, meniscus treatment, cartilage injury, ACL graft type, and activity during injury). Boldface *P* values indicate statistically significant difference between groups compared (*P* < .05). ACL=anterior cruciate ligament; ACLR=anterior cruciate ligament reconstruction; BMI=body mass index; ES, effect size; LCL=lateral collateral ligament; LM=lateral meniscus; MCL=medial collateral ligament; MD=mean difference; PLC=posterior lateral corner.

Supplemental Table S2. Baseline Characteristics of the Included Patients Before Index ACLR

Variable	Primary ACLR (n=19,769)	r-ACLR (n=760)	mr-ACLR (n=13)	r-ACLR vs mr-ACLR: *P* Mean (95% CI) ES
**Age at surgery (y)**	29.1 ± 10.6<br>26 (16-74)	23.0 ± 7.8<br>20 (16-56)	22.6 ± 6.5<br>21 (16-36)	0.94<br>0.365 (-3.385 to 5.308)<br>0.047
**Sex (male)**	10,436 (52.8)	394 (51.8)	7 (53.8)	>0.99<br>2.0 (-27.9 to 29.5)<br>0.04
**BMI (kg/m²)**	24.7 ± 3.3<br>24.3 (15.1-47.1)	24.5 ± 3.0<br>24.2 (17.9-37.6)	24.5 ± 2.6<br>23.6 (21.2–29.4)	0.97<br>-0.024 (-1.547 to 1.783)<br>0.008
**Activity during injury**				0.49
Alpine/skiing	3,464 (17.6)	74 (9.7)	1 (7.7)	>0.99<br>-2.1 (-9.9 to 31.6)<br>0.07
Pivoting sport	12,492 (63.3)	601 (79.2)	10 (76.9)	>0.99<br>-2.3 (-36.3 to 15.5)<br>0.05
Non-pivoting sport	861 (4.4)	21 (2.8)	0 (0.0)	>0.99<br>-2.8 (-4.9 to 29.1)<br>0.24
Other physical activity	795 (4.0)	22 (2.9)	0 (0.0)	>0.99<br>-2.9 (-5.1 to 29.1)<br>0.24
Traffic	326 (1.7)	11 (1.4)	1 (7.7)	0.37<br>6.2 (-1.2 to 39.6)<br>0.30
Other	1,787 (9.1)	30 (4.0)	1 (7.7)	0.83<br>3.7 (-3.8 to 37.2)<br>0.16
**Cartilage injury**				
Lateral femoral condyle	1,122 (5.7)	28 (3.7)	0 (0.0)	>0.99<br>-3.7 (-6.0 to 28.5)<br>0.28
Medial femoral condyle	3,677 (18.6)	118 (15.59)	2 (15.4)	>0.99<br>-0.1 (-13.3 to 34.0)<br>0.00
Lateral patella	661 (3.3)	15 (2.0)	0 (0.0)	>0.99<br>-2.0 (-4.0 to 29.9)<br>0.20
Medial patella	1,047 (5.3)	26 (3.4)	0 (0.0)	>0.99<br>-3.4 (-5.8 to 28.5)<br>0.27
Lateral tibial plateau	1,360 (6.9)	34 (4.5)	0 (0.0)	>0.99<br>-4.5 (-7.0 to 27.7)<br>0.31
Medial tibial plateau	1,059 (5.4)	21 (2.8)	1 (7.7)	0.63<br>4.9 (-2.6 to 38.6)<br>0.22
Trochlea	663 (3.4)	12 (1.6)	0 (0.0)	>0.99<br>-1.6 (-3.5 to 30.3)<br>0.18
**Collateral ligament injury**				
MCL	34 (0.2)	40 (5.3)	1 (7.7)	>0.99<br>2.4 (-5.2 to 35.7)<br>0.10
LCL	171 (0.9)	6 (0.8)	0 (0.0)	>0.99<br>-0.8 (-2.4 to 31.1)<br>0.13
**PLC injury**	34 (0.2)	1 (0.1)	0 (0.0)	>0.99<br>-0.1 (-1.6 to 31.6)<br>0.05

Variable	Primary ACLR (n=19,769)	r-ACLR (n=760)	mr-ACLR (n=13)	r-ACLR vs mr-ACLR: *P* Mean (95% CI) ES
**Meniscus injury and treatment**				
Lateral meniscus injury	4,874 (24.7)	213 (28.0)	3 (23.1)	0.97<br>-4.9 (-22.5 to 29.4)<br>0.11
Lateral meniscus treatment				0.90
Lateral meniscus repair	641 (3.2)	23 (3.4)	0 (0.0)	>0.99<br>-3.0 (-5.2 to 28.9)<br>0.25
Lateral meniscus resection	3,346 (16.9)	145 (19.3)	2 (15.4)	>0.99<br>-3.7 (-16.8 to 30.7)<br>0.10
Lateral meniscus repair + resection	53 (0.3)	2 (0.3)	0 (0.0)	>0.99<br>-0.3 (-1.7 to 31.7)<br>0.07
Lateral meniscus injury left in situ	834 (4.2)	43 (5.5)	1 (7.7)	>0.99<br>2.0 (-5.6 to 35.6)<br>0.08
Medial meniscus injury	5,196 (26.3)	168 (22.1)	0 (0.0)	0.080<br>-22.1 (-26.1 to 11.7)<br>0.75
Medial meniscus treatment				NA
Medial meniscus repair	1,167 (5.9)	33 (4.4)	0 (0.0)	>0.99<br>-4.3 (-6.9 to 27.7)<br>0.30
Medial meniscus resection	3,284 (16.6)	111 (14.6)	0 (0.0)	0.26<br>-14.6 (-18.2 to 18.6)<br>0.58
Medial meniscus repair + resection	32 (0.2)	1 (0.2)	0 (0.0)	>0.99<br>-0.1 (-1.6 to 31.6)<br>0.05
Medial meniscus injury left in situ	713 (3.6)	23 (2.7)	0 (0.0)	>0.99<br>-3.0 (-5.2 to 28.9)<br>0.25
**ACL graft type**				0.92
Patellar tendon autograft	977 (5.0)	34 (4.6)	0 (0.0)	>0.99<br>-4.6 (-7.0 to 27.6)<br>0.31
Semitendinosus autograft	18,129 (93.0)	687 (93.3)	13 (100.0)	0.82<br>6.7 (-25.4 to 9.6)<br>0.38
Quadriceps tendon autograft	300 (1.5)	11 (1.5)	0 (0.0)	>0.99<br>-1.5 (-3.3 to 30.4)<br>0.17
Allograft	56 (0.3)	4 (0.5)	0 (0.0)	>0.99<br>-0.5 (-2.1 to 31.3)<br>0.10
**Direct suture/synthetic/other**	39 (0.2)	1 (0.1)	0 (0.0)	>0.99<br>-0.1 (-1.5 to 31.6)<br>0.05
**Time from injury to surgery (mo)**	20.5 ± 37.0<br>8.2 (0-551)	11.1 ± 22.4<br>5.3(0-316.2)	20.0 ± 46.9<br>5.4 (1.2-167.3)	0.20<br>-8.89 (-15.84 to 12.05)<br>0.387

Data are reported as mean ± SD and median as well as minimum and maximum for continuous variables and as n (%) for categorical variables. The sums may vary because of missing values (variables with missing values were BMI, time from injury to surgery, meniscus treatment, cartilage injury, ACL graft type, and activity during injury). ACL=anterior cruciate ligament; ACLR=anterior cruciate ligament reconstruction; BMI=body mass index; ES = effect size; LCL=lateral collateral ligament; LM=lateral meniscus; MCL=medial collateral ligament; mr-ACLR = multiple revision anterior cruciate ligament reconstruction; NA = not applicable; PLC=posterior lateral corner; r-ACLR=revision anterior cruciate ligament reconstruction.
